# Nitroso Diels-Alder Cycloadducts Derived From *N*-Acyl-1,2-dihydropyridines as a New Platform to Molecular Diversity

**DOI:** 10.3390/molecules25030563

**Published:** 2020-01-28

**Authors:** Andrea Menichetti, Francesco Berti, Mauro Pineschi

**Affiliations:** Department of Pharmacy, University of Pisa, Via Bonanno 33, 56126 Pisa, Italy; andreamenichetti3@msn.com (A.M.); francescoberti86@gmail.com (F.B.)

**Keywords:** diversity oriented synthesis, nitroso Diels-Alder, heterobicyclic compounds, functionalized piperidines, functionalized pyrroles

## Abstract

This review focuses upon the use of nitroso Diels–Alder reactions as a structural complexity generating reaction that has been so far a quite scarcely treated topic, despite its potential. In particular, the use of *N*-acyl-1,2-dihydropyridines as a non-symmetrical diene component in nitroso Diels–Alder reactions encompasses an initial diversification of pathways giving rise to different cycloadducts (direct and inverse). Selective elaborations of these cycloadducts, basically using a reagent-based approach, deliver a discrete number of structurally diverse compounds, including some original heterobicyclic scaffolds and functionalized heterocycles. This forward synthetic planning allowed the individuation of a new biologically active compound based on a novel oxadiaza-bicyclic-[3.3.1]-nonene scaffold which is still under preclinical evaluation.

## 1. Introduction

**Scheme 1:**
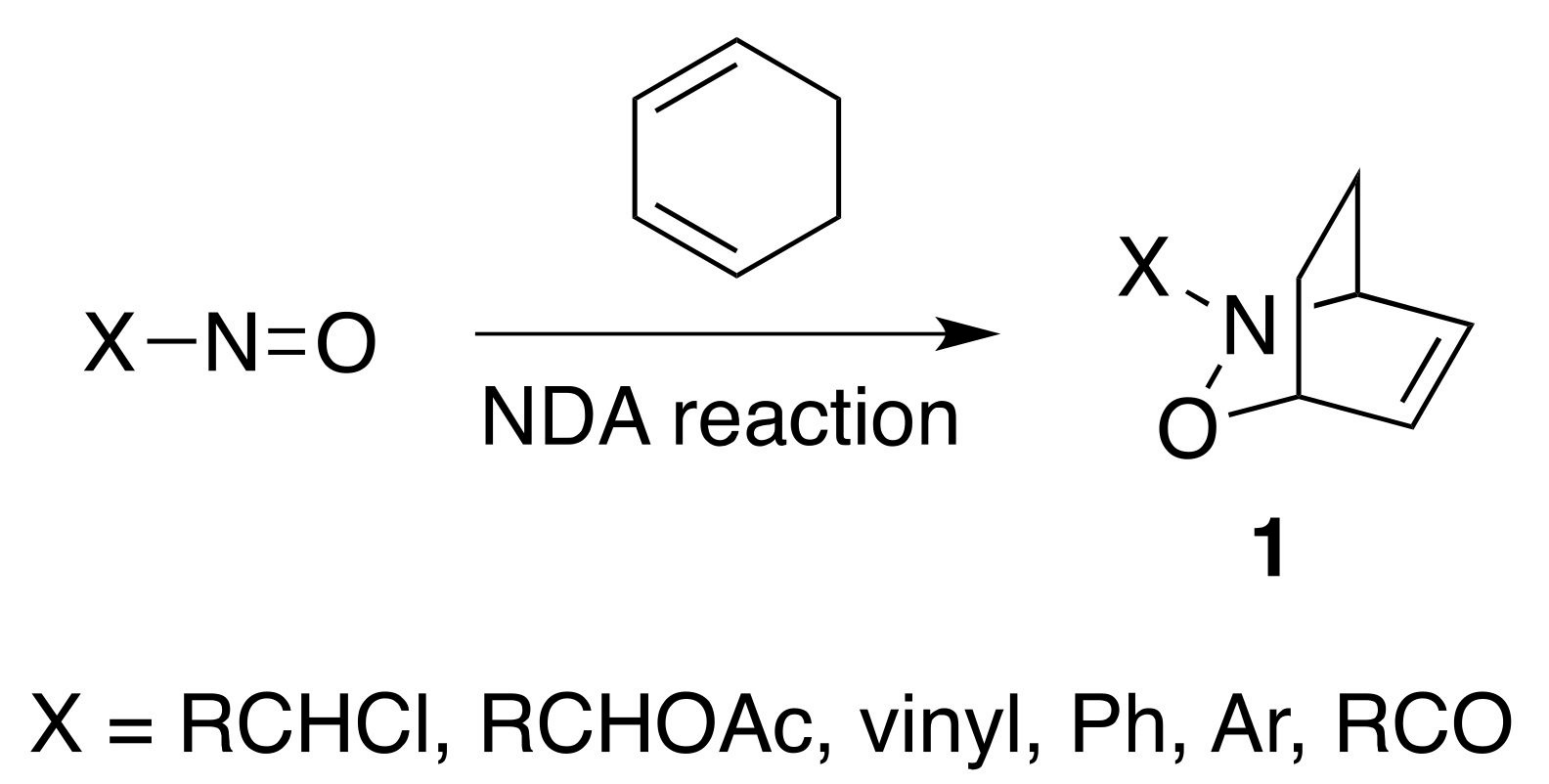
Examples of C-nitroso compounds and of a nitroso Diels–Alder (NDA) reaction.

Organic reactions that result in the formation of multiple covalent bonds in a regio- and stereoselective fashion are very powerful tools in synthetic organic chemistry. Nitroso Diels–Alder (NDA) reactions belong in their own right to this kind of organic transformations, as they are able to install simultaneously a C−O and a C−N bond in a predictable regio- and stereoselective way [1,2,3,4]. A variety of substituted-C-nitroso compounds, such as *α*-chloro-, *α*-acetoxy-, vinyl-, aryl- and acylnitroso, have been widely used as dienophiles in cycloaddition reactions to produce differently substituted 3,6-dihydro-1,2-oxazines of type 1 in a single step (Scheme 1). 

Since the discovery of nitrosobenzene [5], arylnitroso compounds were among the first to be used in NDA reactions. Some of these are stable and commercially available compounds. 

Nevertheless, the direct nitrosation of aromatic compounds is restricted to electron-rich systems, while electron-poor aromatic rings are excluded from this synthetic route. A valuable alternative was described by Taylor and co-workers reporting the preparation of synthetically useful 2-nitrosopyridine starting from 2-aminopyridine [6]. On the other hand, acylnitroso are a very reactive species with a lifetime in the order of 1 ms in organic solution [1,7]. Therefore, they are commonly generated in situ from the corresponding hydroxamic acid by means of periodate salts [8], or by various combinations of transition metals and reoxidating agents [9,10,11]. It is worth mentioning that the possibility to induce asymmetry in the NDA reaction is very much limited. In a seminal paper, Yamamoto reported the first example of a copper–Segphos-catalyzed enantioselective NDA reaction of 6-methylnitroso pyridine and cyclic 1,3-dienes [12]. Later, Studer and Jana reported a highly enantioselective regiodivergent NDA reaction using 2-nitrosopyridine [13]. More recently, Yamamoto and coworkers showed that also the particular chelating properties of pyrimidine and pyrazine arylnitroso derivatives allows the catalytic asymmetric arylnitroso DA reaction to occur with even higher enantioselectivities [14]. An interesting organocatalyzed enantioselective NDA reaction of (non-chelating) nitrosoaryl derivatives has been described by Masson and co-workers [15]. On the other hand, catalytic asymmetric acylnitroso DA reactions have met with very limited success [16,17].

**Scheme 2:**
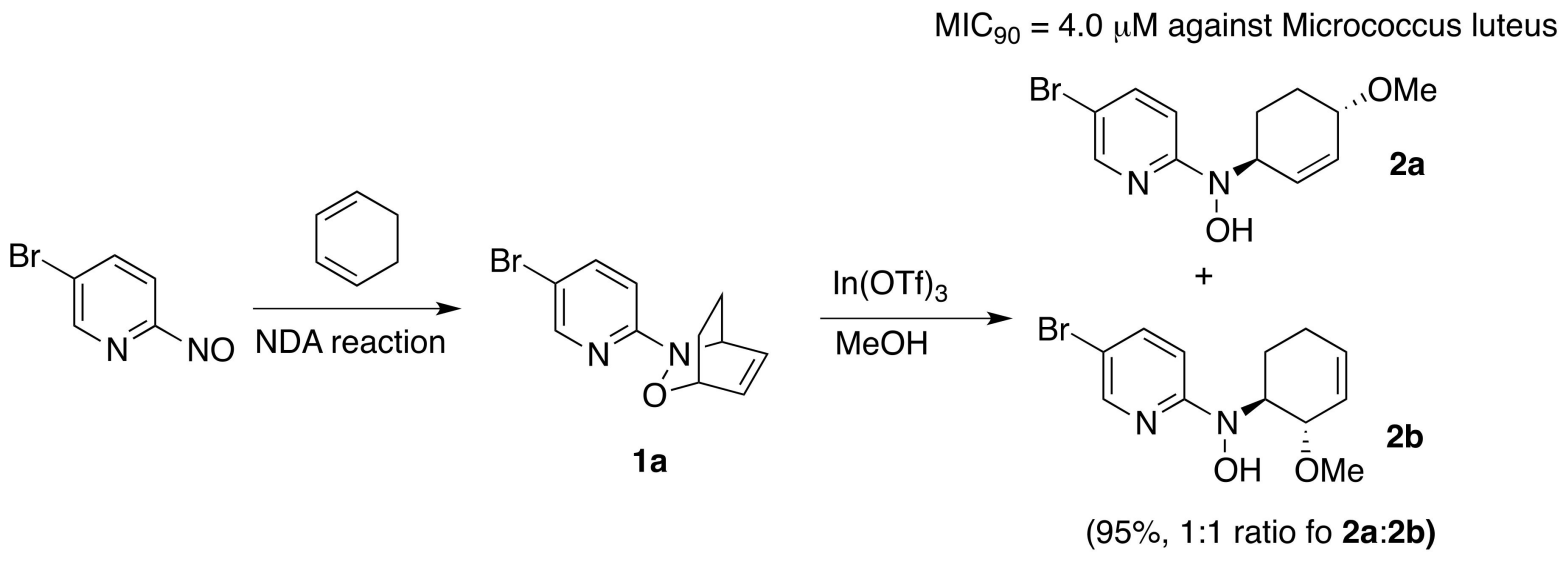
Diversity Oriented Synthesis (DOS) approach for the synthesis of new antibiotics using NDA chemistry by Miller.

Diversity Oriented Synthesis (DOS) has emerged as a powerful strategy in drug discovery, as it allows us to produce structurally diverse and unbiased collections of small molecules covering unique chemical space [18]. The scaffold or skeletal diversity is the most difficult type of chemical diversity to achieve, but also the most attractive one, as the scaffold diversity is a tight requisite for an efficient interaction with biological macromolecules [19]. The search for new molecular entities endowed with biological activity is a fundamental topic in modern drug discovery programs. In fact, the number of new, small molecules launched in the drug market dramatically decreased in recent years, and the application of DOS is considered as a new paradigm for the revival of a drug discovery process centered on small molecules. Despite the rich chemistry of NDA reactions, its clear-cut inclusion in a DOS approach to discover new chemical entities which are possibly useful in medicinal chemistry is an underdeveloped field. To our knowledge, the only explicit DOS approach making use of NDA chemistry has been reported by Miller and co-workers some years ago [20]. They prepared several highly functionalized hydroxylamine-containing compounds using Lewis acid-mediated nucleophilic ring-opening reactions of NDA cycloadducts, such as **1a** with alcohols. Biological screening revealed selective and potent antibacterial activity against *Micrococcus luteus* ATCC 10240 for hydroxylamine compounds with a pyridyl heterocycle such as compounds **2a** and **2b** (Scheme 2).

It should be noted that the use of *N*-acyl-1,2-dihydropyridines (**3**) as the diene component in the NDA reaction and the subsequent elaborations of the obtained cycloadducts have received scarce attention to date. 

**Scheme 3:**
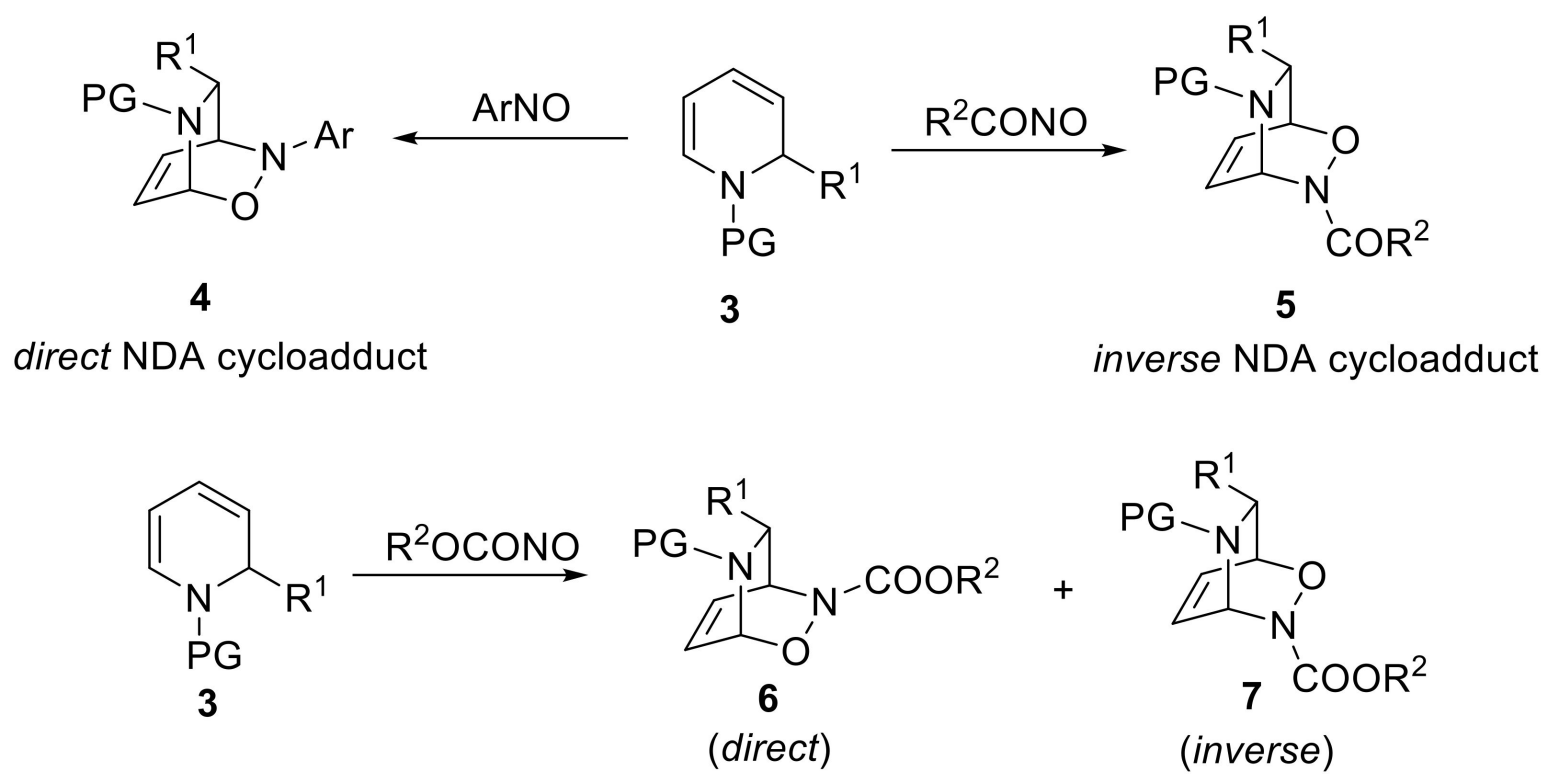
Regioselectivity of NDA reactions of *N*-acyl 1,2-dihydropyridines using diverse nitroso species.

In general, the synthesis of 2-substituted *N*-acyl-1,2-dihydropyridines can be readily accomplished by the nucleophilic addition of carbon nucleophiles to N-acyl-pyridinium salts [21]. The regioselectivity of the reaction has been rationalized in terms of the HSAB model, where soft organometallic reagents, such as organocuprates, generally prefer the C_4_ position, where harder nucleophiles attack the C_2_ position. Unsubstituted *N*-acyl-1,2-dihydropyridines can be regioselectively obtained by a Fowler’s type reduction of the *N*-carbalkoxypyridinium ion using NaBH_4_ [22]. The benefit of NDA reaction that makes use of *N*-acyl-1,2-dihydropyridines relies on a regio- and stereoselective introduction of new C−O and C−N bonds on a pyridine scaffold. It is amply recognized that pyridines, partially hydrogenated pyridines and piperidines are privileged scaffolds in medicinal chemistry [23]. Therefore, the resulting NDA cycloadducts are in principle valuable compounds that can easily be elaborated towards the preparation of substituted partially hydrogenated pyridines. Notwithstanding this encouraging premise, there are only a couple of papers describing the fundamental aspects of this chemistry using acylnitroso dienophiles that date back to the 1980s [24,25]. A very important difference between 1,2-dihydropyridines and normal all carbon-containing 1,3-dienes, is that the former is an inherently dissymmetric diene and the regioselectivity of the cycloaddition can be simply modulated, changing the nature of the group bearing the nitroso moiety. Thus, an arylnitroso species gives with complete regioselectivity the direct cycloadduct of type **4**, whereas very electron-poor acylnitroso species give the inverse bicycle of type **5** with an opposite regioselectivity (Scheme 3). A mixture of both direct and inverse cycloadducts **6** and **7** is obtained when nitrosoformate esters are employed as dienophiles. The observed regioselectivity has been rationalized in terms of FMO theory, considering that *N*-acyl-1,2-dihydropyridine is a particular diene composed of a double bond conjugated with an enamide, thus giving a remarkable asymmetry of the HOMO of the diene [26].

**Scheme 4:**
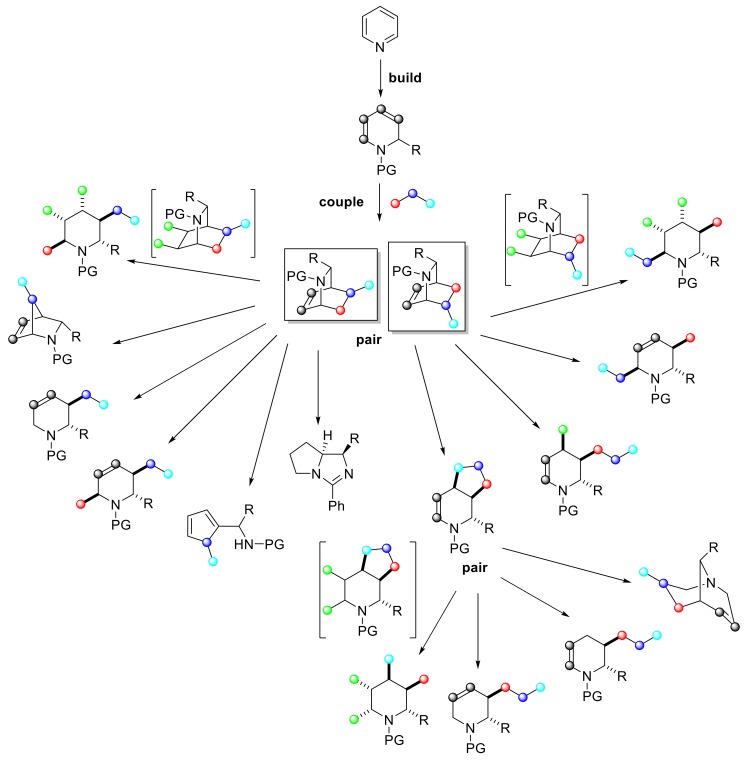
Schematization of B/C/P strategy in NDA chemistry starting from 1,2-dihydropyridines.

The possibility to switch the regioselectivity by a simple change of the nature of the nitroso dienophile is particularly meaningful within a DOS approach as it allows researchers to diversify the couple stage (Scheme 4) at will. Fairly, a conceptualization as a DOS approach of most of the chemistry described in this review was envisioned only a posteriori by the authors. Anyway, we are now providing this kind of B/C/P framework of the NDA reaction starting from *N*-acyl-1,2-dihydropyridines because it offers a clearer picture of the overall strategy, and can offer in perspective further points of the functionalization of the cycloadducts so far not explored.

The development and examples of applications of DOS approach using NDA chemistry starting from 1,2-dihydropyridines will be explained in the following sections. The present Review of DOS approaches using NDA cycloadducts starting from 1,2-dihydropyridines is classified by the type NDA cycloadducts.

## 2. Discussion

### 2.1. Direct NDA Cycloadducts

Arylnitroso species are bench-stable compounds that generally require mild reaction conditions in the NDA reaction with dienes. On the other hand, the resulting NDA adduct might be poorly stable because it is prone to undergo a retro NDA reaction. The rich chemistry of NDA adducts mostly relies on the elaboration of the oxazine moiety, as the cleavage of the N−O bond provides access to 1,4-aminoalcohol derivatives [27]. Having a lower stability than a carbon–carbon bond, the N−O bond can be cleaved exploiting different approaches, such as a combination of zinc and acid [28], molybdenum hexacarbonyl [29,30], titanium(III) salts [31,32], samarium diiodide [33] and metal-catalyzed hydrogenation [34]. 

**Scheme 5:**
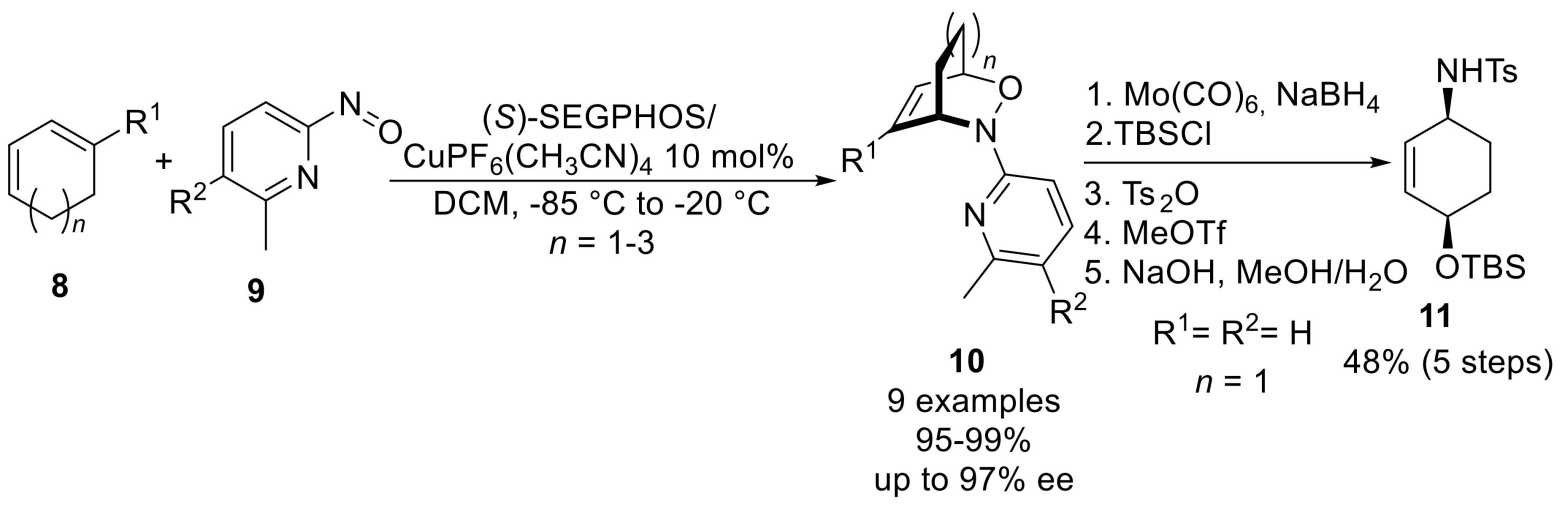
Enantioselective NDA reaction and subsequent *N−O* cleavage.

In addition, a further drawback of the use of arylnitroso is the difficult removal of the aryl moiety. Yamamoto has addressed this issue by using substituted nitroso compounds derived from pyridine [12], pyrimidine and pyridazine [14]. For example, the synthesis of chiral bicycle **10** was ensured by a chelating control of Cu(I) salt exerted on both the phosphine ligand and nitroso pyridine **9** species. After the N−O bond cleavage, the pyridine ring was treated with methyl triflate, and the resulting quaternary intermediate was removed using sodium hydroxide to deliver protected amino-alcohol **11** in enantiomerically-enriched form and satisfactory overall yield (Scheme 5).

**Scheme 6:**
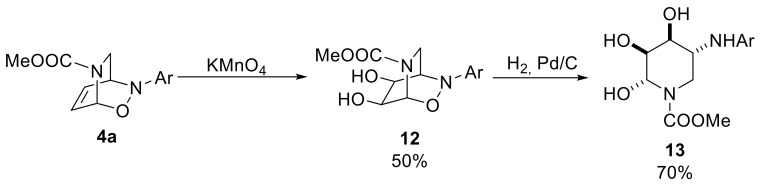
Synthesis of iminosugar derivatives by Streith.

According to the DOS approach proposed in Scheme 4, the elaboration of direct NDA cycloadducts of 1,2-dihydropyridines is the pair stage in which the critical point of diversification is the cleavage of the N−O bond. The 1,4-aminoalcohol framework obtained is installed in a piperidine scaffold in a stereodefined fashion. Moreover, the initial cleavage of the N–O bond unveils a labile N-acyliminium ion precursor that can be properly activated to undergo inter- and intramolecular nucleophilic additions. Hence, the direct NDA cycloadducts elaborations can be classified as a reagent-based approach. In a seminal example, aiming at a stereoselective synthesis of iminosugars, Streith and coworkers reported the first elaboration of such direct NDA cycloadducts [34]. Initially, adduct **4a** was treated with KMnO_4_ to provide the dihydroxylated compound **12** (Scheme 6). The cis-dihydroxylation took place at the opposite face with respect to the N–O bond in a completely stereoselective fashion. The iminosugar **13** was obtained after a palladium-catalyzed reduction of the N–O bond. 

**Scheme 7:**
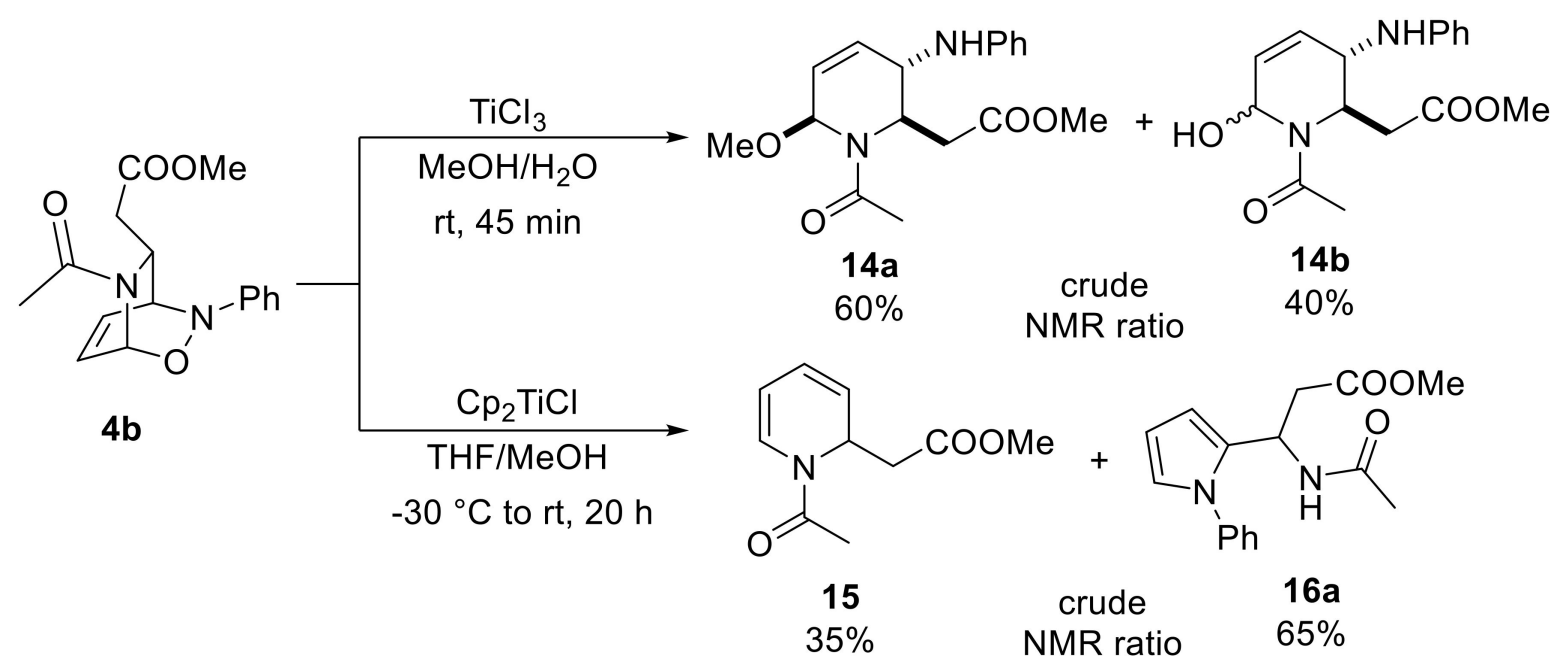
Reagent-based diversification of direct cycloadduct **4b**.

A direct cleavage of the N−O bond without a preventive elaboration of the double bond was reported by Pineschi and co-workers using titanium(III) salts [35]. In particular, exposing the adduct **4b** to TiCl_3_ gave rise to a mixture of *gem*-amino-ether and *gem*-amino-alcohol derivatives **14a** and **14b**, respectively, in which the relative stereochemistry of hydroxyl moiety is lost (Scheme 7). According to the authors, the expected product was not isolated because the titanium species readily catalyzes the conversion of hemiaminal **14b** into a *N*-acyliminium ion amenable to being trapped by the nucleophilic protic solvents. Moreover, **14a** and **14b** are also *N*-acyliminium ion precursors, and an equilibrium of these two species cannot be ruled out. Surprisingly, replacing TiCl_3_ with titanocene monochloride led to a different reaction outcome as 1,2-dihydropyridine **15** and the pyrrole **16a** were recovered. While the formation of 1,2-dihydropyridine **15** was ascribable to a rapid retro NDA process, the synthesis of pyrrole **16** was completely unexpected, and it thus required an extensive study to understand the reaction mechanism.

**Scheme 8:**
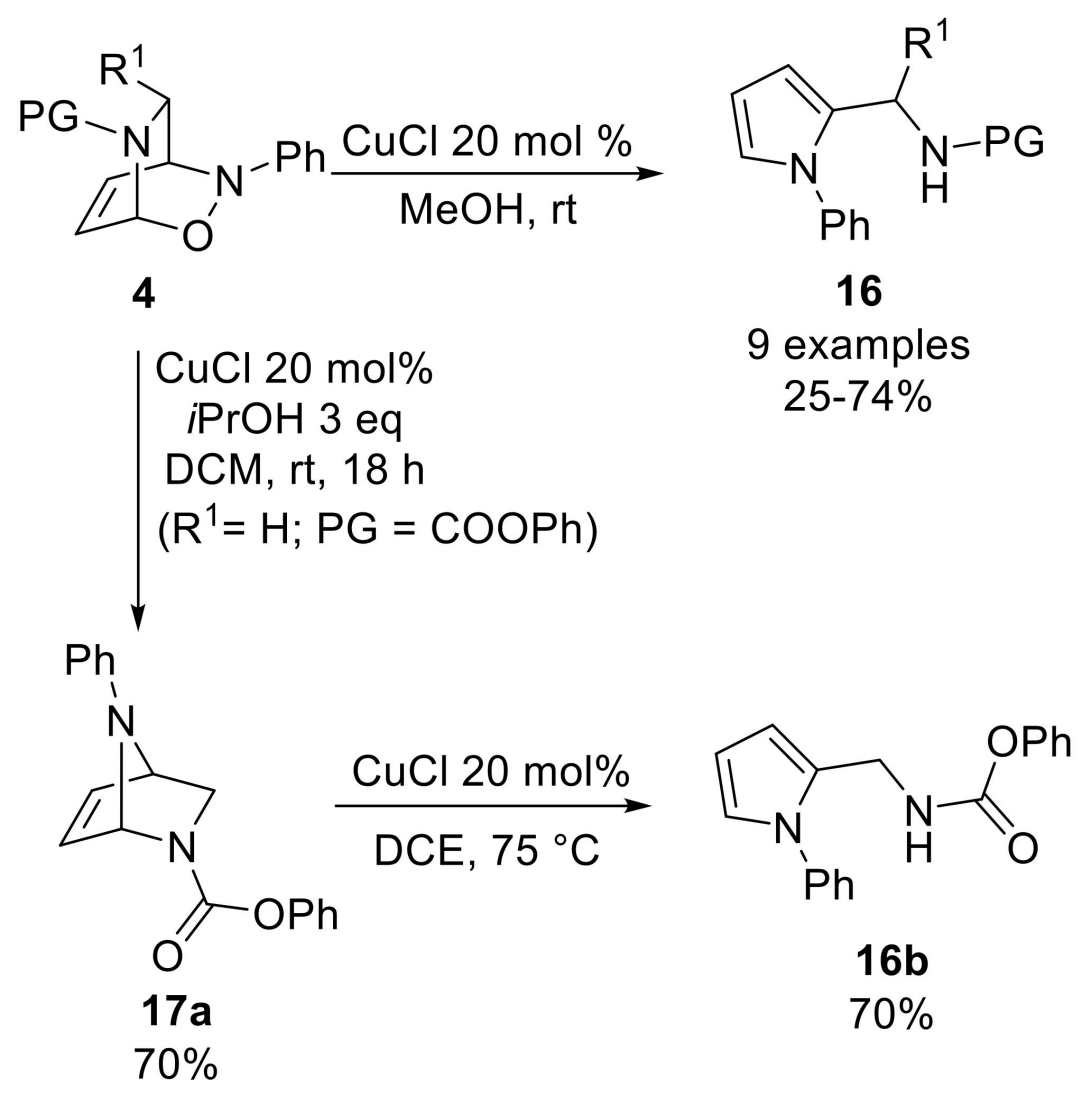
Copper-catalyzed elaborations of direct cycloadducts of type **4**.

A screening of various reaction conditions highlighted that a sub-stoichiometric amount of copper(I)chloride smoothly catalyzed the conversion of cycloadducts **4** into the corresponding pyrrole derivatives of type **16** (Scheme 8). The scope of the reaction was mainly limited to *N*-acyl-protected bicycles, and it could be also carried out in a one-pot, two-step fashion, starting from the appropriately protected 1,2-dihydropyridine. In contrast, sulfonyl-protecting groups showed to be reactive, only raising the temperature while carbamate-protected bicycles gave a lower yield due to the formation of by-products. Nevertheless, reducing the amount of alcohol and using DCM as the solvent enabled the synthesis of a novel [2.2.1]-bicycle **17a** starting from the *N*-phenoxycarbonyl cycloadduct [36]. Moreover, warming **17a** in the presence of copper(I)chloride gave the corresponding methylamino-substituted pyrrole **16b**, suggesting that **17a** is a key intermediate in the pyrrole synthesis.

**Scheme 9:**
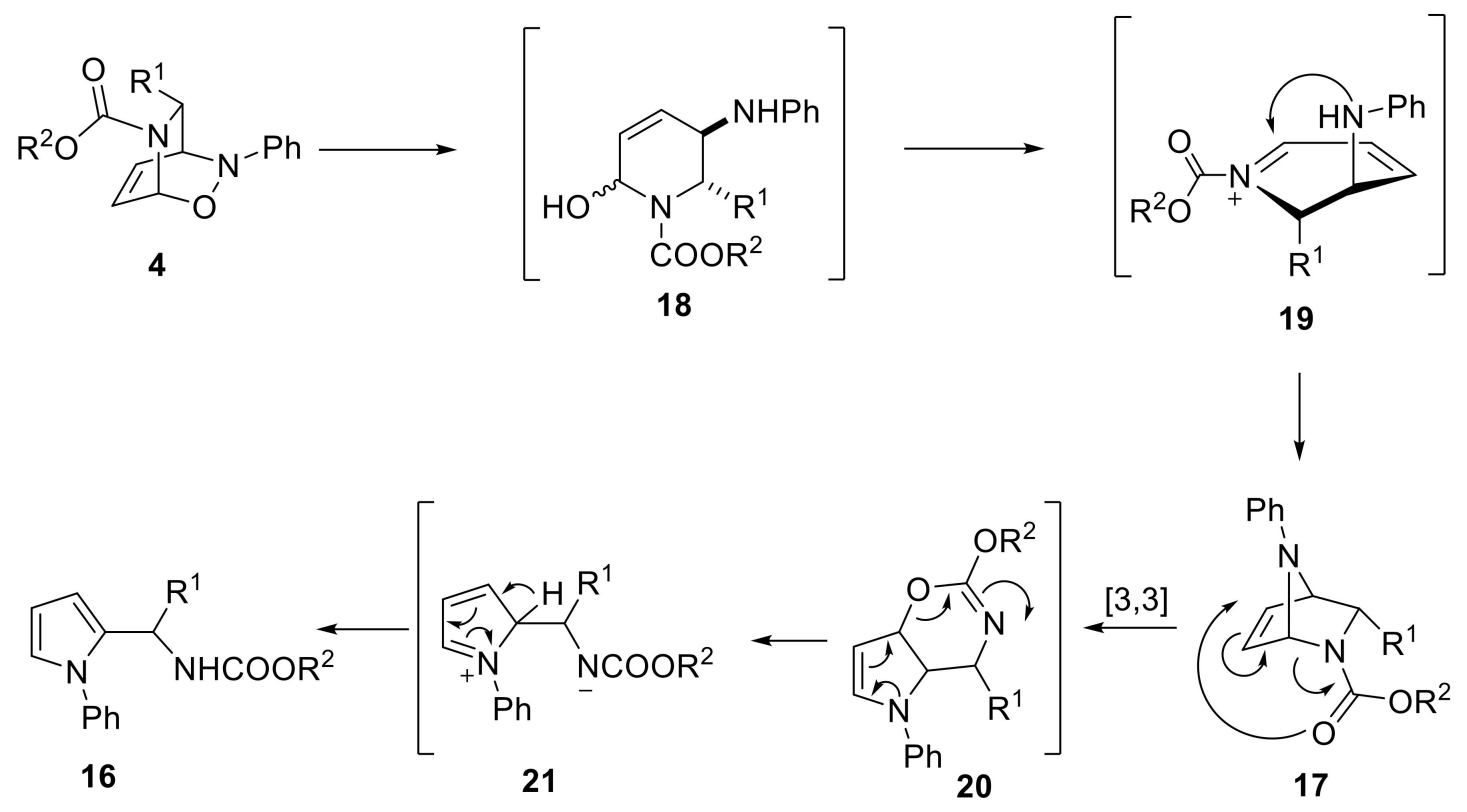
Plausible mechanism for *N*-phenylpyrrole formation.

The proposed reaction mechanism involves a copper-catalyzed cleavage of the N−O bond followed by the formation of a conjugated *N*-acyliminium ion **19**. Subsequent intramolecular addition of the amine moiety gave rise to the 2,7-diazabicycloheptene **17** (Scheme 9). To address the transformation of **17** into the final pyrrole, the author postulated that a [3,3] hetero-Cope rearrangement occurred. At this point, the assistance of elevated temperature is critical when the scaffold bears a carbamate protecting group. 

In fact, in literature, it is known as to the tendency of acyl-protected NDA bicycles to undergo a Cope rearrangement, whereas the bicycles bearing a carbamate moiety are poorly reactive [24]. The last step of the mechanism proceeds via an electronic reorganization of the unstable oxazine **20** to afford **16**. 

**Scheme 10:**
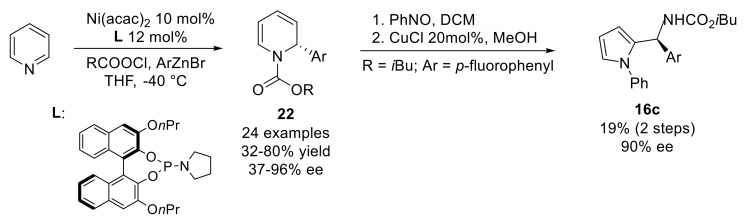
Synthesis of *non*-racemic *N*-phenylpyrroles by Doyle.

It is worth noting that this procedure can be also used to prepare chiral *N*-phenyl pyrroles **16c** (Scheme 10). The chiral center introduced in the starting *N*-carbamoyl-1,2-dihydropyridine **22** is maintained through the synthesis of both the corresponding arylnitroso NDA bicycle and pyrrole adduct [37]. This catalytic asymmetric arylation of pyridine devised by Doyle can be considered as a reliable entry to *non*-racemic 1,2-dihydropyridines used as starting material at the couple stage.

**Scheme 11:**
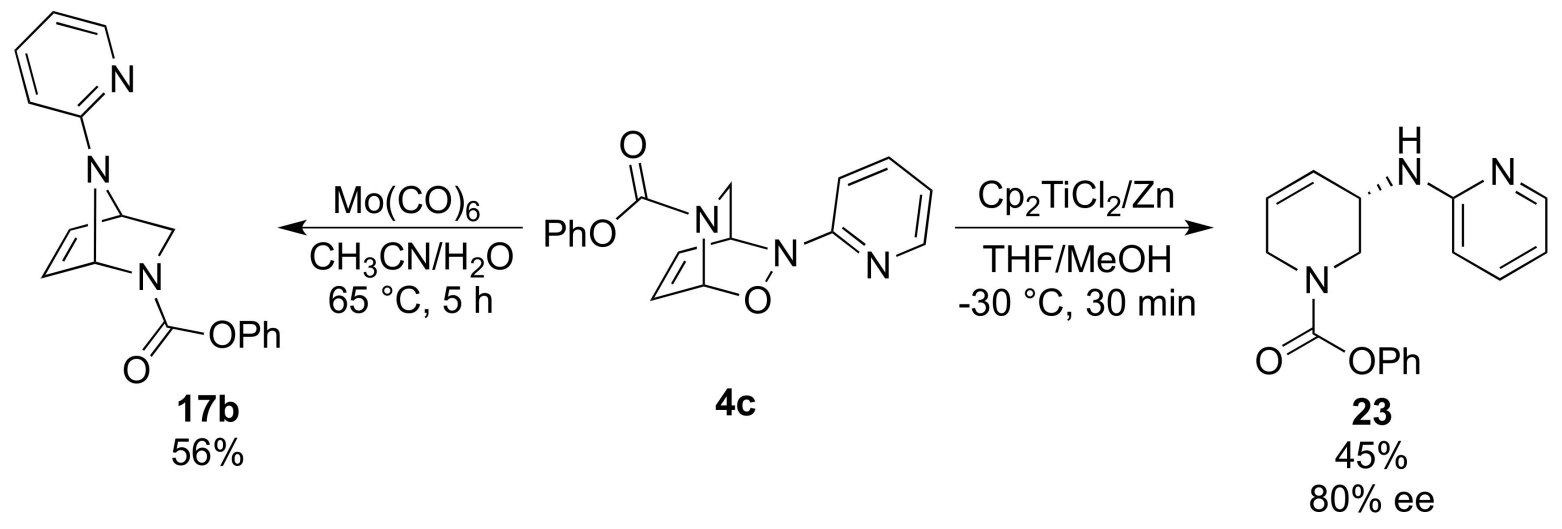
Diversification of pyridine nitroso cycloadduct **4c**.

A further diversification of arylnitroso NDA adducts was achieved by replacing the phenyl moiety with a pyridine [36]. The aforementioned copper-catalyzed approach was unsuccessful in this case, likely due to the formation of a stable complex between the metal and the pyridine subunit. In contrast, alternative known procedures to cleave the N−O bond resulted in the formation of enantio-enriched 1,2,3,6-tetrahydropyridine **23** and [2.2.1] bicycle **17b**. The latter was obtained using Mo(CO)_6_ in a mixture of water and acetonitrile, while upon treatment of **4c** with in-situ-formed titanocene monochloride led to compound **23**. In this case, the initial cleavage of the N−O bond is followed by the reduction of the hemiaminal moiety (Scheme 11).

**Scheme 12:**
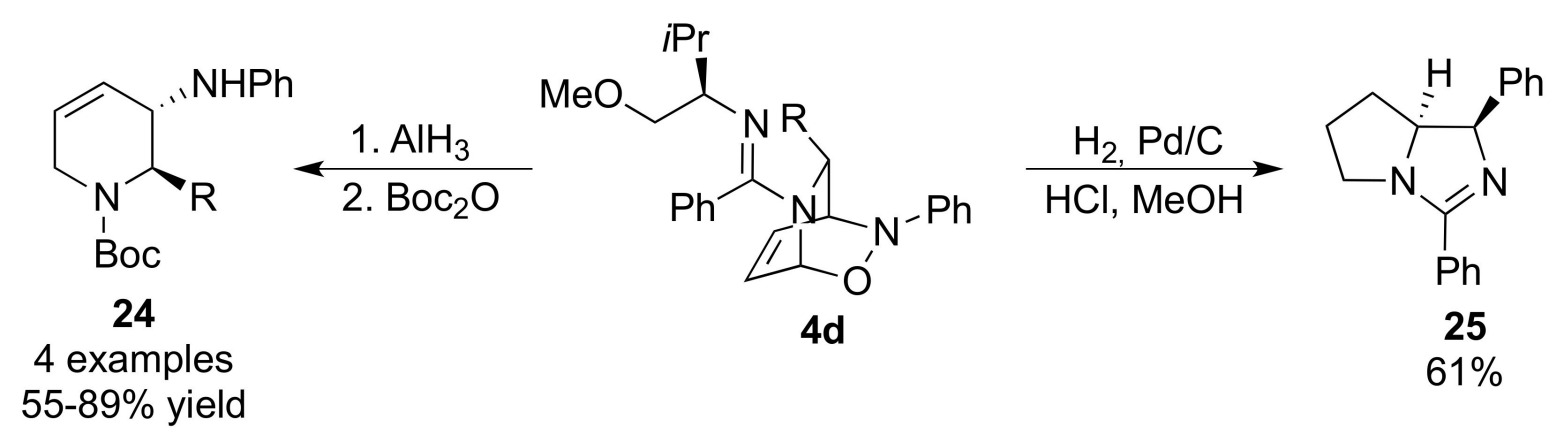
Diversification of chiral non-racemic direct arylnitroso cycloadducts by Charette.

The synthesis of chiral 3-aminopiperidines using NDA adduct was also described by Charette and co-workers [38]. The chiral adduct **4d** was treated with aluminum hydride (Scheme 12), and surprisingly, a chemoselective reduction of the N−O bond, the amidine protecting group and the hemiaminal moiety occurred. The resulting 2,3-disubstituted 4,5-dehydropiperidine was protected using Boc_2_O to afford chiral *non*-racemic **24** with a good yield. An unexpected product was obtained by reducing the NDA adduct **4d** with hydrogen over palladium on charcoal. Bicycle **25** was recovered with a satisfactory yield, although partial racemization occurred.

### 2.2. Elaboration of Inverse Cycloadducts

**Scheme 13:**
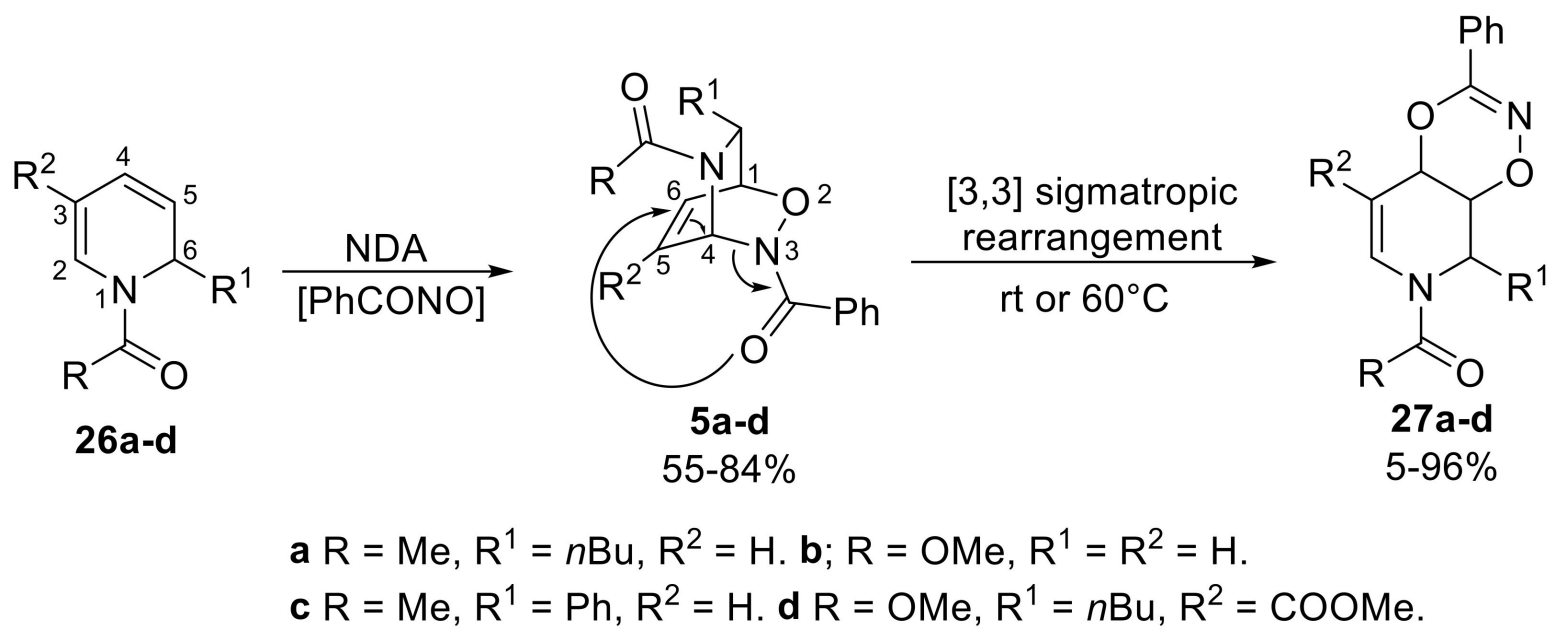
Acyl-NDA reaction and subsequent sigmatropic rearrangement observed by Knaus.

As aforementioned, NDA reactions on 1,2-dihydropyridines with acylnitroso compounds gives inverse cycloadducts selectively, furnishing N-amide-protected ones. On the other hand, carbamate-protected inverse NDA cycloadducts can be obtained in a pure state only by the chromatographic separation of the regioisomeric direct/inverse mixture delivered by the NDA reaction of 1,2-dihydropyridines with carbamoylnitroso compounds [24]. As illustrated in this section, inverse cycloadducts have an interesting capability of diversification in the pair stage in a DOS approach, and several diverse and pharmaceutically relevant molecular skeletons, such as polyfunctionalized tetrahydropyridines and piperidines, iminosugars, dioxazines and unusual oxadiaza[3.3.1]bicycles have been prepared in accordance with a reagent-based approach (see Scheme 4). It should be noticed that inverse NDA cycloadducts have been so far scarcely explored substrates. A seminal in-depth study of the reactivity of inverse cycloadduct was carried out by Knaus and co-workers in 1985 [24]. First of all, both **5a** and **27a** were obtained after chromatographic purification of the crude of NDA reaction, suggesting the possibility of a cycloaddition reaction occurring either on the C_3_–C_6_ or on the C_3_–C_4_ position of 1,2-dihydropyridine (Scheme 13). Nevertheless, a slow conversion of **5a** to **27a** was noticed at room temperature, clearly demonstrating that dioxazine **27a** is not the primary reaction product. Also, cycloadducts **5b** and **5c** slowly underwent conversion to **27b** and **27c** at room temperature, while only heating up to 60 °C in dimethyl sulfoxide (DMSO), compound **5d** afforded complete conversion to **27d**. The authors proposed a reasonable [3,3]-sigmatropic rearrangement pathway to explain the obtained results. Importantly, only benzoyl–nitroso cycloadducts (**5a**–**d**) with very few substitution patterns were examined, whereas carbamate–nitroso cycloadducts proved to be unreactive. This behavior was ascribed to the lower electron-withdrawing effect of the carbonyl attached to N_3_, which is not sufficient electron-withdrawing to trigger the C_4_–N_3_ shift necessary for the rearrangement reaction.

**Scheme 14:**
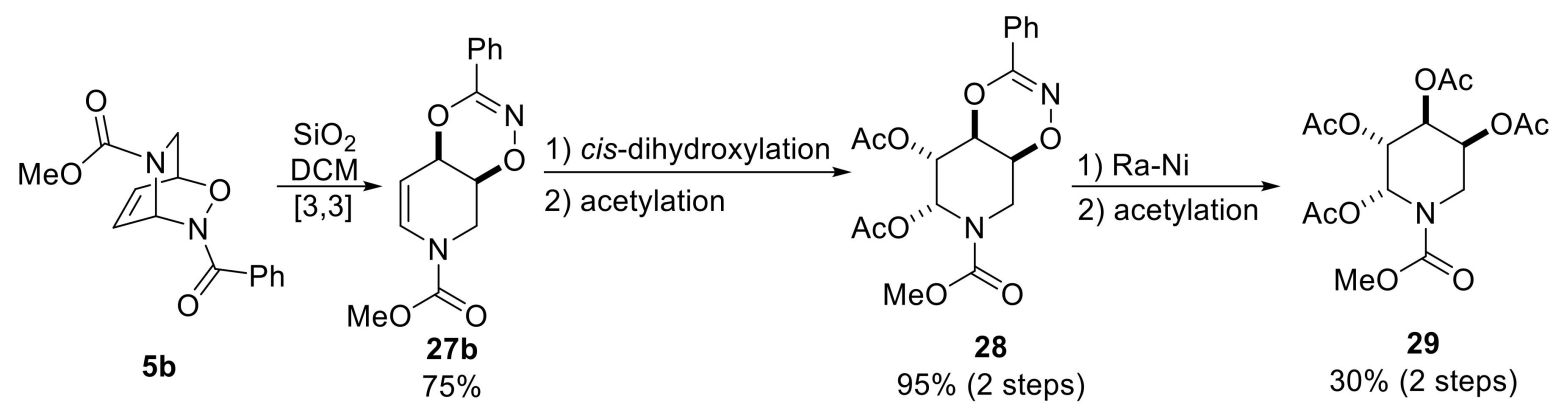
Dioxazine diversification en route to iminosugars.

The formation of a dioxazine ring by [3,3]-sigmatropic rearrangement was also reported by Streith and co-workers in 1990 [39]. However, the corresponding experimental procedure was very poorly defined, and the authors simply indicated that the treatment of a dichloromethane solution of NDA cycloadduct **5b** with a large amount of silicic acid afforded stereospecifically the dioxazine **27b**. Aiming to synthesize iminosugar derivatives (Scheme 14), the authors performed a *cis*-dihydroxylation of the latter compounds that occurred *anti* with respect to the dioxazine backbone to give orthogonally-protected polyhydroxylated piperidines **28** (diacetate). The subsequent reductive destruction of the dioxazine ring with Raney nickel delivered, after peracetylation, the tetraacetate **29** which was an arabinose derivative. Hence, in addition to an elaboration of the NDA bicycle, the authors carried out a manipulation on the dioxazine system allowing the obtainment of an analogous-carbohydrate scaffold, that can be considered the first example of a pair stage on the dioxazine backbone in a general concept of DOS (see above, Scheme 4).

**Scheme 15:**
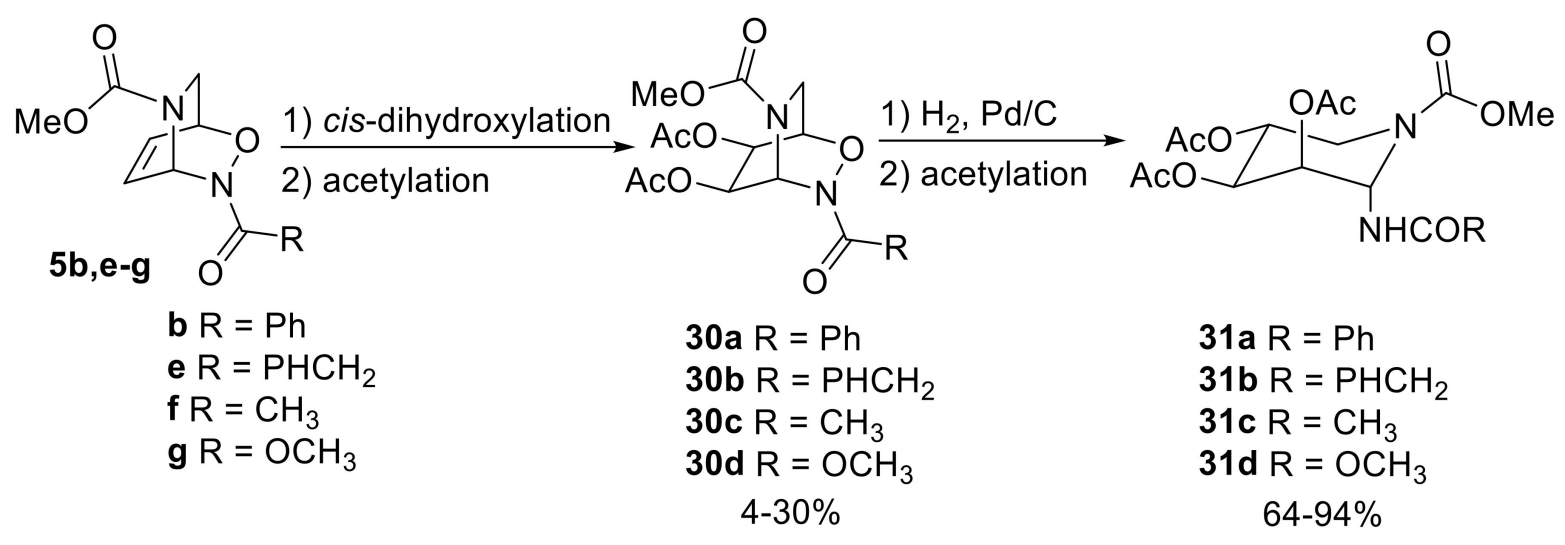
Acyl-NDA diversification en route to iminosugars.

Some years before, the same research group developed a further diversification of NDA cycloadducts to deliver racemic iminosugars [25]. They initially performed the *cis*-dihydroxylation (and subsequent acetylation) of some inverse cycloadducts (**5b**, **e**–**g**). As this reaction occurred in a stereospecific manner exclusively in anti with respect to the N−O bridge, the following reductive hydrogenolysis (and acetylation) allowed the formation of racemic amino-*α*-dl-lyxopyranose derivatives **31a**–**d** (Scheme 15).

**Scheme 16:**
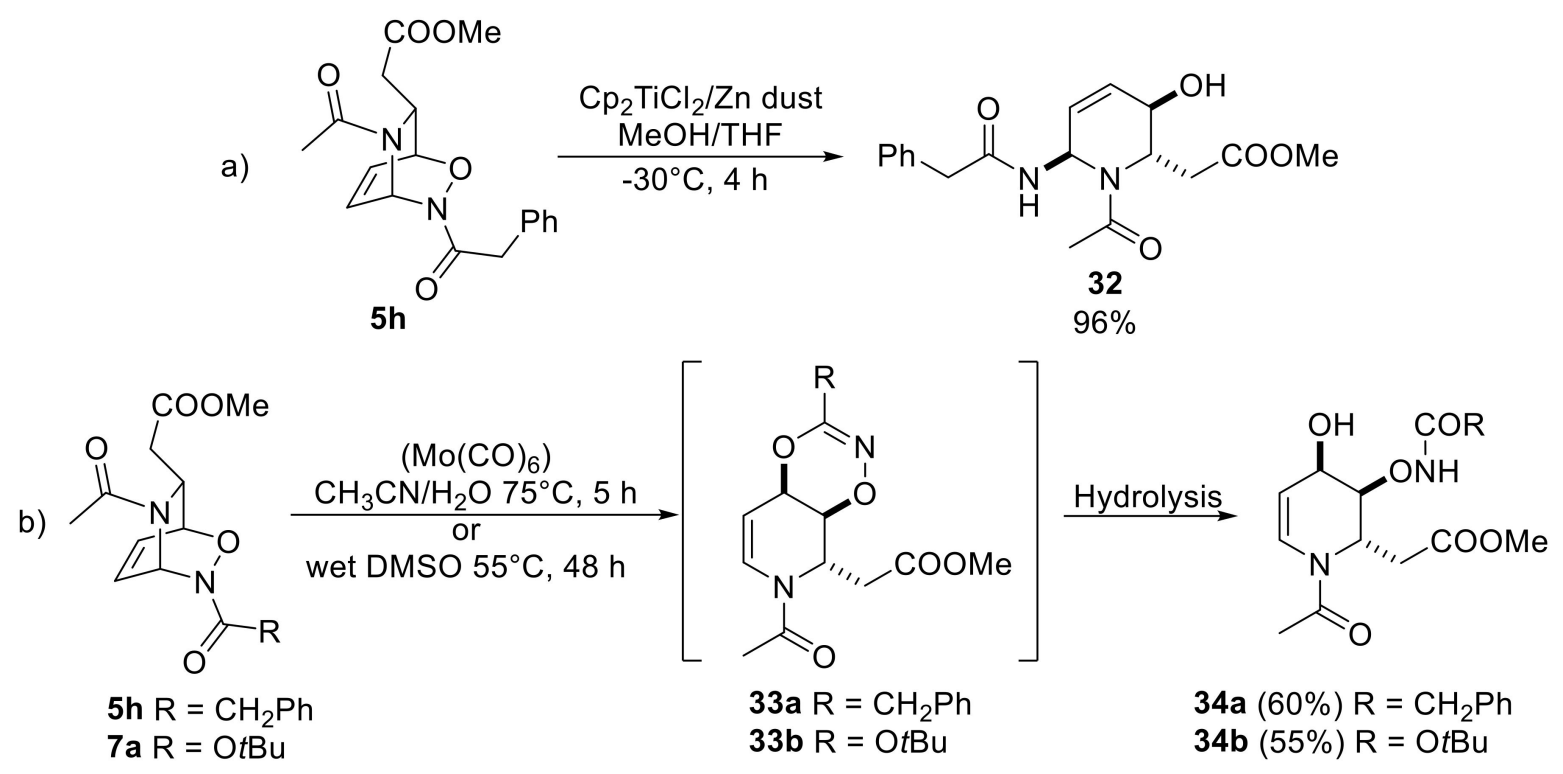
Different outcomes of the reductive treatment of acyl-NDA cycloadducts. Conditions **a**: cleavage of the N−O bond. Conditions **b**: [3,3]-rearrangement.

To find other elaborations of inverse cycloadducts, we need to move about thirty years after, when the Pineschi and co-workers group attempted N−O cleavage protocols directly on inverse cycloadducts [40]. Amide-protected substrate **5h** was subjected to N−O cleavage by the use of stoichiometric amount of Cp_2_TiCl_2_ [32], yielding *gem*-diamine iminosugar **32** in an efficient fashion (Scheme 16a). It should be noted that *gem*-diamine iminosugars represent an important class of glycomimetic compounds possessing biological activity [41]. In the same study, the formation of a dioxazine system (**33a**, **b**) was postulated as intermediate (Scheme 16b) for the formation of an endocyclic enecarbamate motif. 

In fact, the use of Mo(CO)_6_ at 75 °C in a mixture of CH_3_CN/H_2_O furnished the unexpected 4-hydroxytetrahydropyridine **34a** and **34b**. After careful analysis of reaction conditions, molybdenum hexacarbonyl was found unnecessary, and the same products, albeit more slowly, were also formed in wet DMSO at 55 °C with both carbamate- and amide-protected cycloadducts. It should be noticed that in these conditions dioxazines **33a**,**b** cannot be isolated as a pure compound but, the formation of observed product inevitably has to come from a [3,3]-sigmatropic rearrangement, that generates a dioxazine intermediate, and its subsequent hydrolysis.

**Scheme 17:**
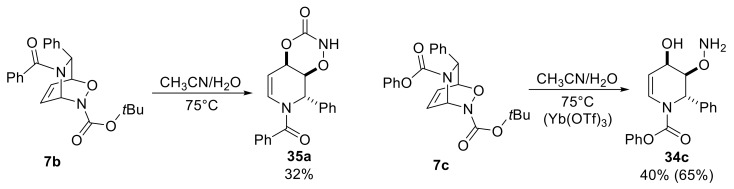
Rearrangement of carbamate-NDA cycloadducts.

Due to the remarkable importance of 4-hydroxytetrahydropyridines in many fields [42], different carbamate nitroso compounds were used in previously mentioned thermal conditions, but the reaction outcomes were found to be not much predictable. Only bicycles **7b** and **7c** were found to be reactive, albeit forming a different type of products (**35a**, **34c**), as shown in Scheme 17, either derived from the hydrolysis processes. Thus, the nature of substituent at the 6-position of starting DHP and also of the nature of the protecting groups showed a considerable influence on the reaction outcomes. In particular, the presence of the phenyl group seemed to be essential to promote the rearrangement for this type of cycloadducts. Importantly, the use of water-tolerated Lewis acid, such as Yb(OTf)_3_, has markedly increased the yield of compound **34c** up to 65%.

**Scheme 18:**
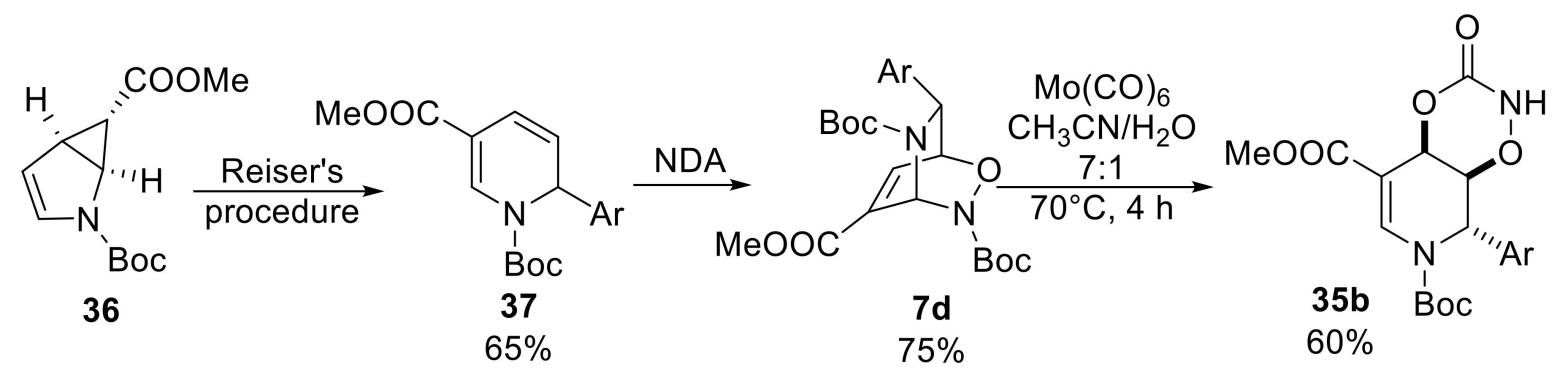
New entries to 1,2-dihydropyridines and subsequent elaboration of the carbamate-NDA cycloadduct.

Very recently, similar reaction conditions were applied on Boc-protected cycloadduct **7d** by Reiser and co-workers, achieving bicyclic enecarbamates **35b** containing an ester moiety at the C_5_ position [43] (Scheme 18). They developed an innovative palladium-catalyzed synthesis of 1,2-dihydropyridines (**37**) starting from monocyclopropanated pyrroles (**36**), and after the NDA reaction, the obtained inverse cycloadducts **7d** underwent a rearrangement reaction with Mo(CO)_6_ and CH_3_CN/H_2_O at 70 °C, providing compounds of type **35b**.

**Scheme 19:**
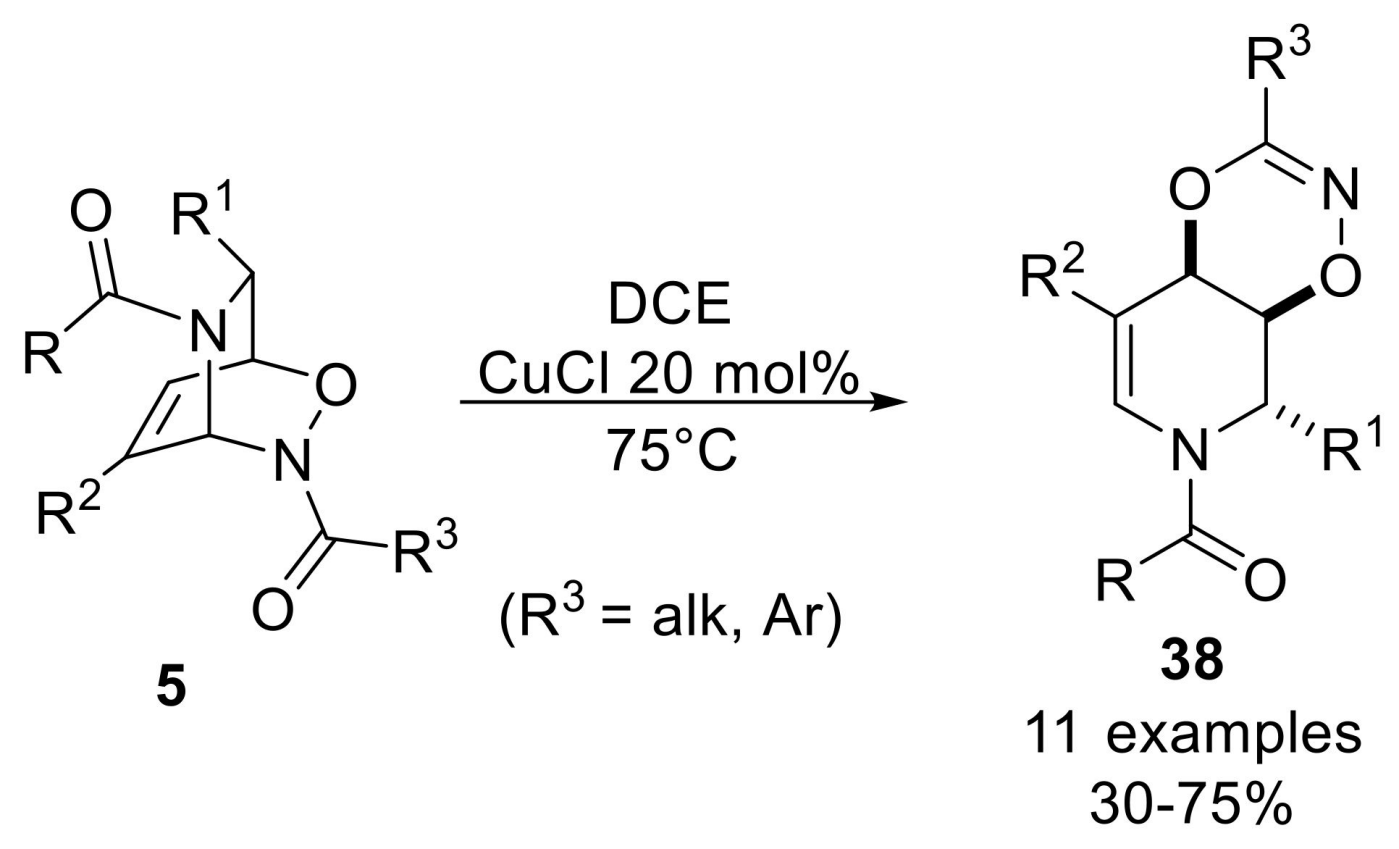
Copper-catalyzed rearrangement reactions of amide-NDA cycloadducts.

The shortage of data on hetero-Cope rearrangement reactions of amide-NDA cycloadducts stimulated Pineschi and co-workers to investigate alternative reaction conditions to prepare dioxazine compounds, since they could be an interesting key intermediate for the synthesis of nitrogen-containing scaffolds [44]. To this end, an extensive screening of reaction conditions was carried out for amide-protected inverse cycloadducts, as only thermal reaction conditions showed very long reaction times and incomplete conversion. The rearrangement took place by means of the use of a catalytic amount of CuCl in a dichloroethane solution of inverse cycloadduct, heating up to 75 °C to give moderate to good yields of dioxazine **38**. It should be noticed that the use of degassed solvent notably increases the reaction rate and the yield of the process (Scheme 19).

**Scheme 20:**
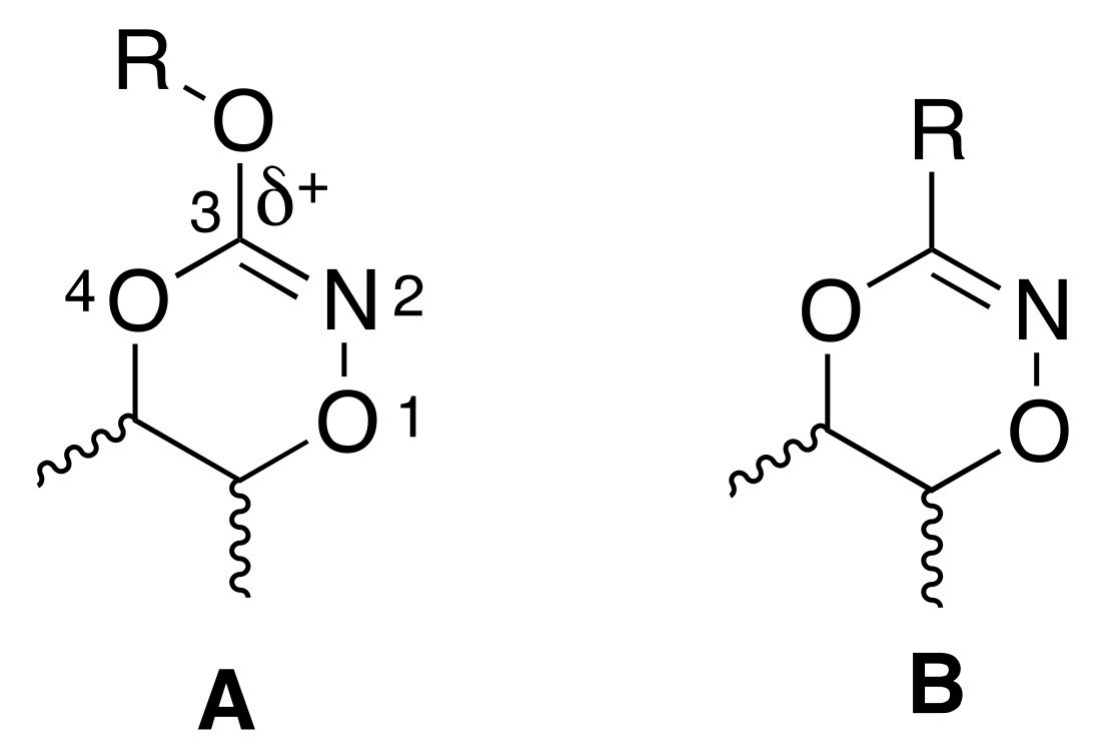
Diverse reactivity of the C_3_ carbon in the carbamate (**A**) and amide (**B**) derived 1,4,2-dioxazines.

Importantly, under these reaction conditions, carbamate-protected cycloadducts turned out to be unreactive. Therefore, an important differentiation of reactivity between carbamate and amide series cycloadducts may lead to achieve a reagent-based diversification in accordance with the concepts of DOS. The different reactivity observed between the NDA adducts of the carbamate and amide series can be ascribed to the different nature of the carbon at the C_3_ position of the 1,2,4-dioxazine skeleton. In fact, the carbamate-derived dioxazine of type **A** has never been isolated as such, but they can be admitted as a transient intermediate in the formation of 4-hydroxypiperidines **34a**–**c** (see Scheme 17 and Scheme 18) [44]. Starting from amide nitroso derivatives (compounds of type **B**) we are dealing with less reactive *O*,*O*-dialkylhydroxamate derivatives, whereas we have a more reactive alkoxyimido carbonate, such as **A** when dealing with carbamate nitroso cycloadducts (Scheme 20). The possibility in the latter to give hydrolysis products is reasonably related with the increased electrophilicity of the C=N double bond in compounds having the **A** framework, as shown by quantum-chemical computations [44].

### 2.3. Reductive Elaboration of 1,4,2-Dioxazines

**Scheme 21:**
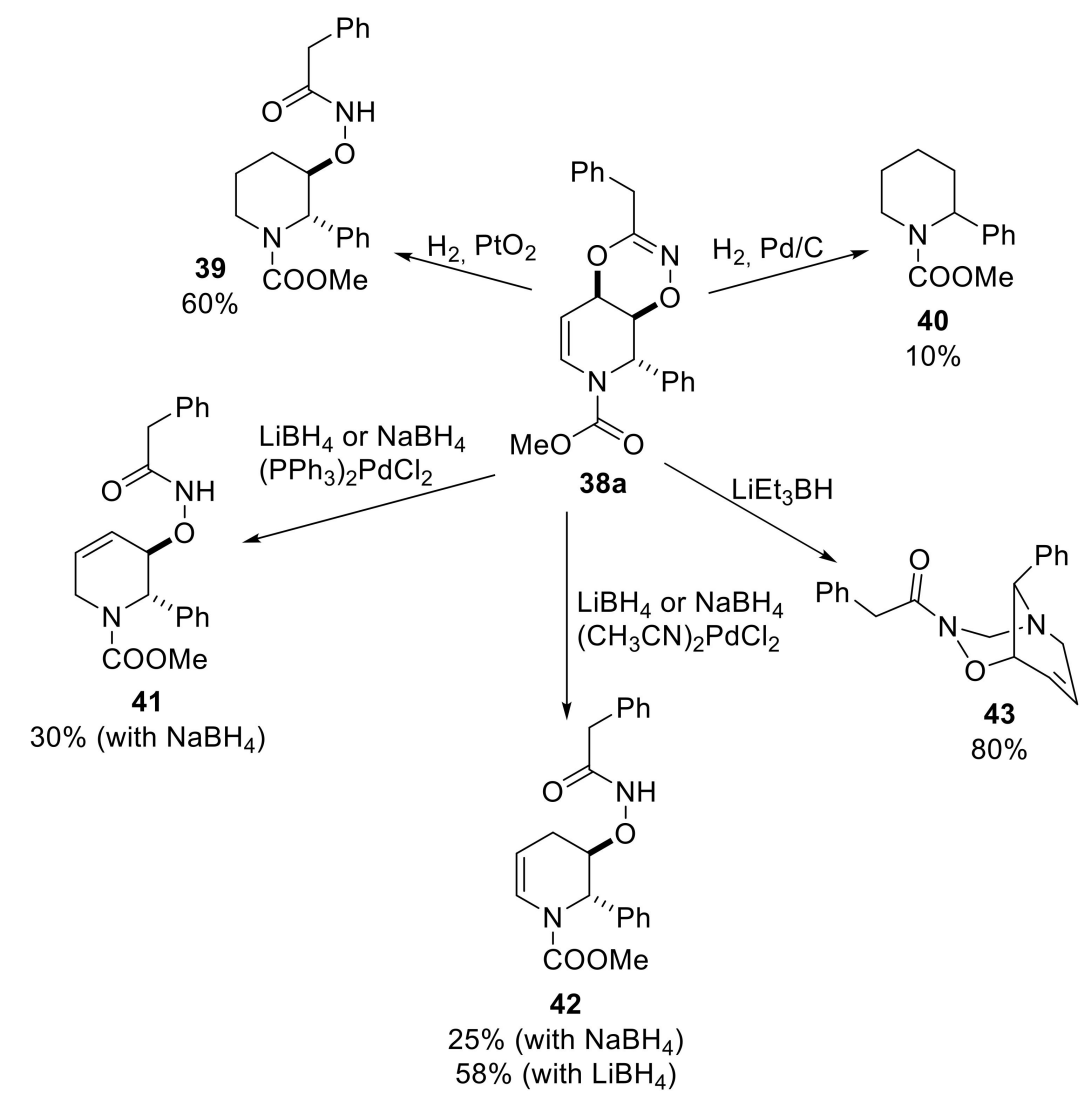
Reductive diversifications of 1,4,2-dioxazine **38a**.

To our knowledge, the first and unique example of a reductive manipulation of a bicyclic 1,4,2-dioxazine have been described in the previous paragraph for the synthesis of iminosugars (see Scheme 14) [39]. Basically, twenty-eight years later, Pineschi and co-workers chanced upon a diversification on the behavior of dioxazines in the presence of different combinations of hydride sources and/or metal catalysts [45]. Some preliminary unpublished results had already shown the different behavior of dioxazines in diverse catalyzed reductive protocols. For example, hydrogenation of compound **38a** in the presence of PtO_2_ furnished selectively the *trans*-disubstituted piperidine **39**, but the use of Pd/C gave also the 2-substituted piperidine **40** as a minor product (70/30 NMR ratio). Then, heterogeneous hydrogenation carried out with ammonium formate at 80 °C with a catalytic amount of Pd/C [46] provided a 60/40 mixture of piperidines **39** (minor) and **40** (major). A plausible reason of this reactivity involves the oxidative addition of palladium or platinum to the enecarbamate moiety, due to the known capacity of these metals to generate π-allyl complexes [47]. The displacement of the dioxazine motif as an unconventional leaving group generates a first 3,4-tetrahydropiperidine intermediate that can undergo a direct reduction of a double bond in the presence of PtO_2_, giving selectively compound **39**, or a second oxidative addition in the presence of palladium, given by the higher propensity of this metal to form π-allyl complexes. The second oxidative addition and subsequent reduction of the double bond delivered piperidine **40** (Scheme 21). Interestingly, 2,3-disubstituted piperidine scaffolds (type **39**) represent a pharmaceutically interesting molecular skeleton, since this motif is included into non-peptidic human neurokinin-1 (NK1) substance P receptor antagonists, natural products, antiprotozoal agent and potential chemotherapeutic agents [48,49,50]. The use of common reducing agents, with or without palladium catalysts, provided selectively Δ^2,3^- or Δ^3,4^-piperidine derivatives **41** and **42** with unconventional substitution patterns [45]. In particular, NaBH_4_ or LiBH_4_ in presence of 5 mol % of (CH_3_CN)PdCl_2_ gave selectively compound **42**. Replacing the palladium catalyst with (PPh_3_)_2_PdCl_2_ leads to a mixture of **41** and **42** in different ratio using NaBH_4_ or LiBH_4_. Moreover, employing a different hydride sources such as LiBEt_3_H (Super Hydride^®^) and NaBEt_3_H reagent in the presence of (CH_3_CN)PdCl_2_ as a catalyst, compound **42** was selectively generated. Serendipitously, an unusual and novel heterobicycle **43** was obtained with high selectivity, making use of (PPh_3_)_2_PdCl_2_ and (PPh_3_)_4_Pd. Furthermore, with Pd(dba)_2_, a 63/37 mixture of **42** and **43** was detected. Importantly, it was noticed that the selective formation of compound **43** can be also achieved in moderate to good yields without any palladium-based catalysts starting from carbamate-protected dioxazines [45].

**Scheme 22:**
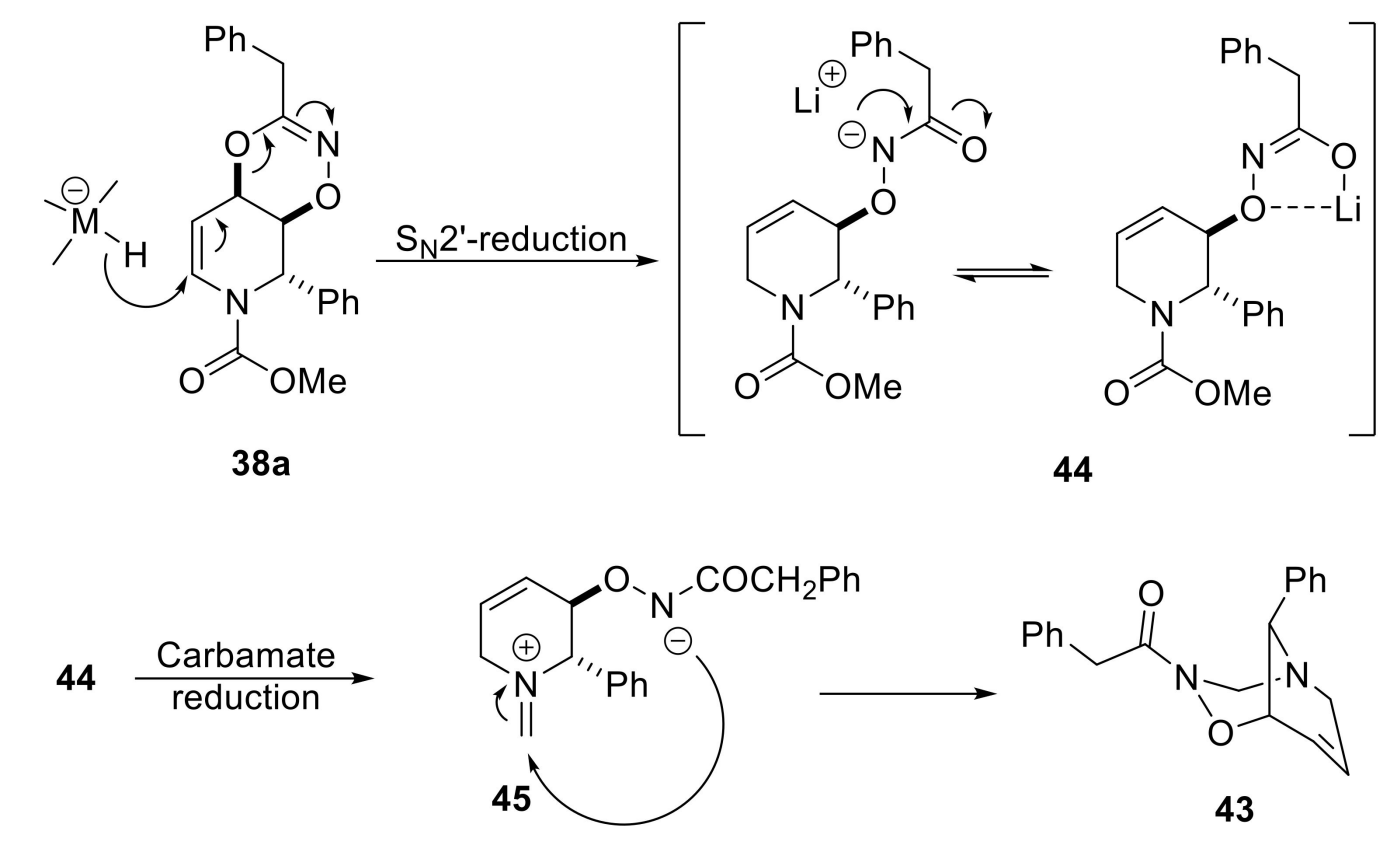
Plausible mechanism for the formation of the 1,3-diaza-4-oxa-[3.3.1]-nonene framework.

The attainment of compound **43** having a novel 1,3-diazo-4-oxa-bicyclic-[3.3.1]-nonene architecture has been fully rationalized by means of a cascade reaction (Scheme 22). The only way to obtain 1,3-diaza-4-oxa-[3.3.1]-bicyclic framework **43** comes from an initial regioselective S*_N_*2′-addition of the hydride to enecarbamate **38a**. The obtained anionic 3,4-unsaturated intermediate **44** does not undergo a further reduction of the oxyamido functionality, arguably due to an intramolecular chelate stabilization. It is also likely that the concomitant reduction of the carbamate moiety of species **44** gives rise to a zwitterionic iminium ion **45** that undergoes intramolecular trapping by *N*-alkylation to deliver the bicyclic framework **43**. In accordance with the proposed mechanism, the formation of such a bicyclic framework necessarily requires the presence of a carbamate protecting group on the endocyclic piperidine nitrogen [45].

**Figure 1:**
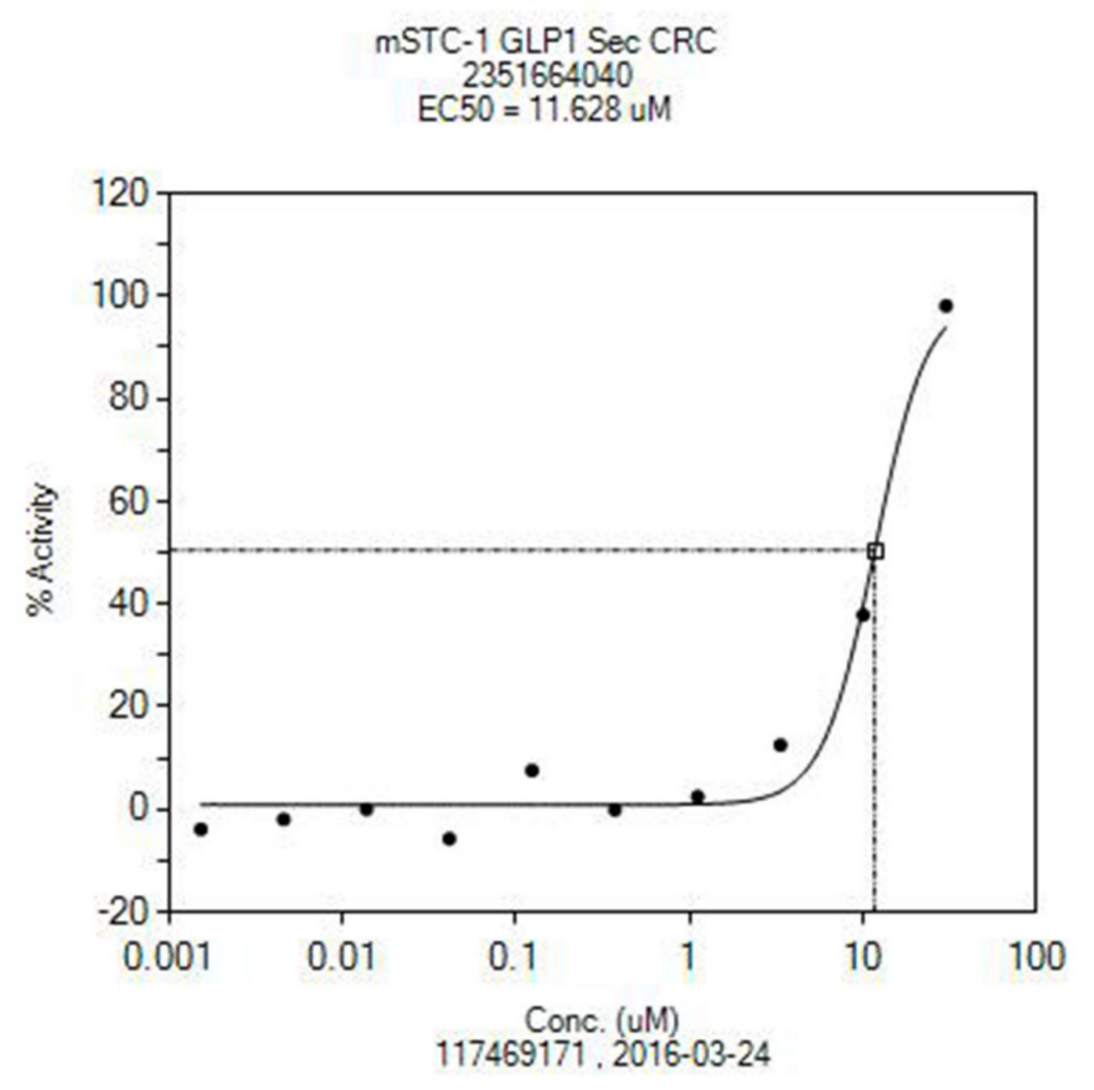
Increase in GLP-1 secretion by compound **43**.

In the Open Innovation Drug Discovering Program sponsored by Eli Lilly, compound **43** showed a promising in vitro activity promoting the release of GLP-1 (glucagon-like peptide 1) (Figure 1). GLP-1 is a 30 amino-acid peptide hormone that triggers insulin secretion in pancreatic cells, and that has proven difficult to activate with small molecules. Existing molecules that stimulate its secretion are only peptides similar to the native GLP-1 peptide, and are delivered by injection. Therefore, there is a huge interest in finding new orally active drugs useful for treatment of diabetes and NAFLD by means of novel mechanisms of action [51].

## 3. Conclusions

In this review, a systematic overview of the possibilities so far offered by the NDA chemistry starting from 1,2-dihydropyridine is provided and rationalized for the first time within a DOS approach. In fact, the reaction of aryl-, acyl- and carbamoyl nitroso derivatives with *N*-protected 1,2-dihydropyridines afford NDA cycloadducts of different kinds that can be differently manipulated, offering a range of synthetic alternatives. 

Most of the diversifications were obtained using different reductive reaction conditions on the NDA cycloadducts in a reagent-based approach to give stereochemical and skeletal diversity. A DOS conceptualization of the NDA chemistry of 1,2-dihydropyridines provided in this review can offer further points of functionalization beyond the stages of diversification so far individuated. For example, nucleophilic ring-opening reactions of NDA cycloadducts with 1,2-dihydropyridines have not yet been explored. Overall, the chemistry herein described is particularly valuable, considering the remarkable importance that partially hydrogenated pyridines and piperidines have in biologically active compounds. In particular, the obtainment of novel sp^3^-rich scaffolds having biological activity can give new inputs in medicinal chemistry programs, with the necessary inclusion on the active scaffold individuated of the less appealing diversity (i.e., appendage diversity), in order to find more potent compounds.
